# Immunological serotype interactions and their effect on the epidemiological pattern of dengue

**DOI:** 10.1098/rspb.2009.0331

**Published:** 2009-04-15

**Authors:** Mario Recker, Konstantin B. Blyuss, Cameron P. Simmons, Tran Tinh Hien, Bridget Wills, Jeremy Farrar, Sunetra Gupta

**Affiliations:** 1 Department of Zoology, University of OxfordOxford OX1 3PS, UK; 2 Department of Engineering Mathematics, University of BristolBristol BS8 1TR, UK; 3 Hospital for Tropical Diseases190 Ben Ham Tu, Quan 5, Ho Chi Minh City, Viet Nam; 4 Oxford University Clinical Research Unit, Hospital for Tropical DiseasesHo Chi Minh City, Viet Nam

**Keywords:** dengue, antibody-dependent enhancement, mathematical model

## Abstract

Long-term epidemiological data reveal multi-annual fluctuations in the incidence of dengue fever and dengue haemorrhagic fever, as well as complex cyclical behaviour in the dynamics of the four serotypes of the dengue virus. It has previously been proposed that these patterns are due to the phenomenon of the so-called antibody-dependent enhancement (ADE) among dengue serotypes, whereby viral replication is increased during secondary infection with a heterologous serotype; however, recent studies have implied that this positive reinforcement cannot account for the temporal patterns of dengue and that some form of cross-immunity or external forcing is necessary. Here, we show that ADE alone can produce the observed periodicities and desynchronized oscillations of individual serotypes if its effects are decomposed into its two possible manifestations: enhancement of susceptibility to secondary infections and increased transmissibility from individuals suffering from secondary infections. This decomposition not only lowers the level of enhancement necessary for realistic disease patterns but also reduces the risk of stochastic extinction. Furthermore, our analyses reveal a time-lagged correlation between serotype dynamics and disease incidence rates, which could have important implications for understanding the irregular pattern of dengue epidemics.

## 1. Introduction

Dengue is caused by infection with any one of the four closely related virus serotypes DEN-1, DEN-2, DEN-3 and DEN-4 (Calisher *et al*. 1989), and transmitted to humans primarily by the *Aedes aegypti* mosquito vector. Its emergence and geographical spread are of major global concern and it has seen a 30-fold increase in incidence during the last 50 years. The virus currently affects over 50 million people annually in the tropical and subtropical regions of the world and causes tens of thousands of deaths (Gubler 2002; WHO 2008). Clinical manifestations of dengue can include dengue fever (DF) and the more severe dengue shock syndrome and dengue haemorrhagic fever (DHF), a life-threatening illness with case fatality rates up to 20 per cent in the absence of treatment (which can be reduced to less than 1 per cent with proper medical management).

Despite recent advances (Edelman 2007), there are currently no licensed vaccines or effective antiviral drugs available against the disease. One of the key problems in developing a vaccine is that pre-existing antibodies to a particular serotype can exacerbate an infection of a heterologous serotype. This effect is hypothesized to be due to the action of antibody-dependent enhancement, or ADE, whereby cross-reactive antibodies elicited by a previously encountered serotype bind to the newly infecting heterologous serotype, but fail to neutralize it (Halstead 1979). The virus–antibody complex engages Fc receptors on the surface of cells such as macrophages and dendritic cells (Takada & Kawaoka 2003), enabling the virus to enter the cell more easily for replication (Rothman & Ennis 1999; Boonnak *et al*. 2008). Animal studies indicate that the ADE process results in higher viral burdens *in vivo*, which is likely to explain the epidemiological association of severe disease with secondary infection by a heterologous serotype (Burke *et al*. 1988; Thein *et al*. 1997; Vaughn *et al*. 2000; Goncalvez *et al*. 2007) and also the association of severe disease in infants with passively acquired antibody (Kliks *et al*. 1988; Chau et al. 2008).

The temporal epidemiological pattern of dengue is characterized by multi-annual fluctuations in DF or DHF incidence and also cyclical oscillations of the individual serotypes whereby the dominant type is sequentially replaced over time (Nisalak *et al*. 2003); it is unclear, however, whether and how these two features relate to one another. Various mathematical models have been proposed to illuminate these intriguing dynamics and to uncover the driving forces behind them. The consequences of the effect of ADE on the transmission dynamics of dengue were studied by Ferguson *et al*. (1999), and it was shown that ADE could promote the coexistence of strains and lead to nonlinear dynamics. Cummings *et al*. (2005) further demonstrated that ADE-driven dynamics can provide a good qualitative approximation to the chaotic desynchronization of strains as commonly observed within longitudinal datasets of numbers of clinical cases (Schwartz *et al*. 2005), although the selective advantage of ADE may be capped by threats to persistence arising from increasingly high amplitude oscillations. However, it has since been argued that either the period of oscillations or the desynchronization between serotypes generated by ADE alone is not in good agreement with those observed among reported time series of DHF cases, and that some form of cross-protective immunity (Wearing & Rohani 2006; Nagao & Koelle 2008) or seasonal forcing (e.g. Adams *et al*. 2006) is necessary to explain the observed epidemic periods and disease patterns.

Here, we present the results of an epidemiological model for dengue transmission, which employs two parameters to represent the effects of ADE. In addition to a parameter describing the enhancement of transmission during secondary infection through increased viraemia, we include the possibility of enhanced susceptibility to heterologous infections among individuals who have experienced primary infections due to the existence of non-neutralizing cross-reactive antibodies. We show that by including both facets explicitly, both the observed temporal patterns of DF outbreaks and serotype dynamics can robustly be reproduced without the need for extrinsic factors such as seasonal variation or stochasticity. The model further allows us to investigate the relationship between the epidemic pattern of dengue and the dynamics of the co-circulating serotypes. Analyses of time series produced by our model suggest that overall incidence rates and relative serotype prevalence, and especially their respective rates of change, are intrinsically linked.

## 2. Material and methods

### (a) Mathematical model

We use a simple mathematical model to investigate the transmission dynamics of four co-circulating dengue serotypes within the population, similar to the one analysed by Cummings *et al*. (2005). We assume that recovery from an infection with any one serotype provides permanent immunity against this particular serotype, but can lead to an enhancement of other serotypes upon secondary infection after which individuals acquire complete immunity against all four serotypes. (The last assumption is based on the rarity of third or fourth dengue infections; e.g. Gibbons *et al*. 2007.) With these assumptions, we divide the population into the following classes: *s* denotes the fraction of the population that has not yet been infected with any of the serotypes and is thus totally susceptible; *y*_*i*_ is the proportion infectious with a primary infection with serotype *i*, *r*_*i*_ is the proportion recovered from primary infection with serotype *i*; *y*_*ij*_ is the proportion infectious with serotype *j*, having already recovered from infection with serotype *i*; and, finally, *r* is the proportion of completely immune (those who have recovered after being exposed to two serotypes). The model equations are given as follows:

$$\begin{array}{c} \text{d}s/\text{d}t=\mu -s\sum_{k=1}^{4}\lambda_{k}-\mu s,\\ \text{d}y_{i}/\text{d}t=s\lambda_{i}-(\sigma +\mu )y_{i},\\ \text{d}r_{i}/\text{d}t=\sigma y_{i}-r_{i}(\mu +\sum_{j\neq i}\gamma_{ij}\lambda_{j}),\\ \text{d}y_{ij}/\text{d}t=r_{i}\gamma_{ij}\lambda_{j}-(\sigma +\mu )y_{ij},\;i\neq j,\\ \text{d}r/\text{d}t=\sigma \sum_{i=1}^{4}\sum_{j\neq i}y_{ij}-\mu r. \end{array}$$

The host population size is assumed to be constant with 1/*μ*(=70 years) being the average host life expectancy and 1/*σ*(=3.65 days) is the duration of infectiousness. As dengue infections lasts less than a week (Kuno 1997), the incidence of multiple simultaneous infections is assumed to be negligible. Within this framework, the force of infection of serotype *i*, *λ*_*i*_, is given by

$$\lambda_{i}=\beta_{i}\left(y_{i}+\sum_{j\neq i}\phi_{ji}y_{ji}\right),$$

where *β*_*i*_(=400 per year) is the transmission coefficient of serotype *i*. The effects of ADE are represented by two parameters that describe (i) the enhancement of susceptibility to secondary infection, *γ*_*ij*_≥1, and (ii) the increase in transmissibility during secondary infection, *ϕ*_*ij*_≥1. The model behaviour can then be analysed by varying the respective degrees of enhancement while keeping all other parameters constant.

### (b) Cross-correlation

To investigate a possible effect of the serotype dynamics on disease prevalence within the population, we used a time-dependent Shannon index of biodiversity

$$H(t)=-\sum_{i=1\ldots 4}p_{i}(t)\text{log}(p_{i}(t)),$$

where *p*_*i*_(*t*) is the relative frequency of serotype *i* at time *t*, and calculated the cross-correlation between the two time series *H*(*t*) and total disease prevalence *P*(*t*)$\left(=\sum_{i,j}y_{i}(t)+y_{ij}(t)\right)$ as follows:

$$r(\Delta )=(P⊗H)(\Delta )=\frac{\sum_{t=1\ldots n}\left(P_{t}-\mu_{P})(H_{t+\Delta }-\mu_{H}\right)}{\sigma_{P}\sigma_{H}},$$

where *μ*_*P*_ and *μ*_*H*_ are the means, and *σ*_*P*_ and *σ*_*H*_ are the standard deviations, of time-series *P* and *H*, respectively; *n* is the number of data points.

### (c) Data

Data on yearly patterns of disease incidence, monthly patterns of dengue hospitalization and relative serotype abundance in southern Viet Nam were kindly provided by the dengue surveillance programme at the Institute Pasteur, Ho Chi Minh City.

## 3. Results

### (a) Dengue epidemiology in southern Viet Nam

As in most countries in South East Asia, dengue is highly endemic in Viet Nam, particularly in the tropical southern regions of the country. Figure 1 shows the epidemiological data of total disease incidence and relative serotype prevalence for South Viet Nam from 1994 to 2007 (serological data were not available for the years 1994 and 1995), together with a more detailed temporal record of children hospitalized with dengue at monthly time intervals for the period 2004–2007. Figure 1*a* clearly demonstrates the two key epidemiological features of dengue: first, the total incidence rates of DF/DHF show marked variations over the years, with no indication of a trend towards either reduced or increased disease burden within this region. Second, the serotype dynamics display the characteristic fluctuations of single-type dominance over time. Interestingly, despite the period covered by the data in figure 1*a* being too short for a meaningful analysis of the inter-epidemic period, it is obvious that it is likely to be longer than the 3-year cycles often reported to underlie dengue incidence rates in Thailand.

Figure 1*b* offers a more detailed picture of dengue epidemiology within this region over a 4-year period, revealing the strong seasonal component in dengue transmission, which peaks between July and October before dropping to very low levels over the drier winter months. Notably, though, there is no pronounced component of seasonal forcing in the longitudinal behaviour of the individual serotypes. We therefore do not take into account seasonal or stochastic variation in dengue transmission, but investigate whether immune interaction through ADE and cross-immunity can cause the cyclical behaviour in disease incidence and serotype prevalence.

### (b) The effects of ADE

To compare the relative impact of ADE on the transmission dynamics of dengue and its four serotypes, we used a simple compartmental model and performed a series of simulations varying the two key model parameters: the enhancement of susceptibility to secondary infection by a heterologous serotype, *γ*, and the increased transmissibility during secondary infection due to elevated viral load, *ϕ* (see §2 for full model description). Figure 2 shows the various time series tracing the level of prevalence of the four serotypes within the population over time in response to changes in *γ* and *ϕ*. The system readily exhibits an abundance of different dynamical behaviours. For small degrees of enhancement (*ϕ*,*γ*<1.2), the system approaches a steady-state equilibrium (figure 2*a*) that is replaced by synchronized oscillations as the level of enhancement increases. Further increase in *γ* or *ϕ* desynchronizes the serotypes, resulting in out-of-phase oscillations of increasing amplitude and periods. As the effects of ADE amplify, we observe prolonged episodes of very low disease prevalence with intermittent epidemic outbreaks (figure 2*i*).

Of particular interest here is the region of chaotic oscillations that most closely resemble the observed temporal pattern of DF/DHF. Within this region, we find the characteristic multi-annual cycles (*π*∼3–5 year) in disease prevalence (see the electronic supplementary material for the according periodograms) and, importantly, the sequential replacement of prevailing serotypes. This behaviour is exemplified in figure 3, which contrasts a typical output of our model with a previously published time record of dengue cases in Thailand (Nisalak *et al*. 2003). The figure shows a good qualitative agreement between the data and model output in both overall serotype dynamics and disease incidence.

We then analysed the model dynamics within the parameter space of enhancement with respect to synchrony between individual serotypes (figure 4*a*), serotype replacement (figure 4*b*), inter-epidemic period (figure 4*c*) and serotype persistence (figure 4*d*). From the time series shown in figure 2, it is clear that the desynchronization of serotypes leads to chaotic fluctuations and the characteristic replacements of the dominant types over time. Studying the synchronization patterns within the (*ϕ*,*γ*)-parameter plane by measuring the divergence between various pairs of serotypes over time, we found that desynchronization and hence chaotic oscillations typify a significant portion of the parameter region (figure 4*a*). Using a measure for single-type dominance similar to the one introduced by Recker *et al*. (2007), we also found that sequential serotype replacement where individual serotypes are temporarily dominant characterize most of the (*ϕ*,*γ*)-parameter space (figure 4*b*). This confirms that desynchronized fluctuations in serotype prevalence are not an artefact of a particular combination of parameter values, but rather the principal model behaviour under the action of ADE alone. Importantly, these oscillations arise even at relatively small values of *ϕ* and *γ*, and for realistic values of the basic reproductive number, *R*_0_; that is, we do not require biologically unreasonable high levels of enhancement (either in transmissibility or susceptibility) to produce a realistic epidemiological and serological pattern of dengue.

The reduction in the degree of enhancement necessary for desynchronized oscillations achieved by the decomposition of the effects of ADE has two important implications for the system's dynamical behaviour. First, it allows for greater flexibility in terms of inter-epidemic periods with a significant proportion of the (*ϕ*,*γ*)-parameter space yielding realistic 3–5 year epidemic cycles in disease incidence (figure 4*c*). Second, the reduction in enhancement also lowers the threat of stochastic extinction due to large amplitude oscillations (as shown in figure 4*d*) as the proportion of time a particular serotype persists above a given threshold level (we chose a threshold of 1E-8, in line with Cummings *et al*. 2005).

### (c) Relationship between serotype and infection dynamics

We next investigated a possible association between disease prevalence and serotype dynamics by measuring diversity *H*(*t*) and total prevalence *P*(*t*) over a 50-year period. Figure 5*a* shows a typical time course of relative serotype frequency generated by our model, with the according time series for disease prevalence, *P*, shown in figure 5*b*. From figure 5*b*, we can identify the presence of a time-lagged correlation between infection prevalence and diversity, in that the fluctuations in prevalence appear to trail the fluctuations in diversity. This time-delayed correlation is even more pronounced between the time courses of the rates of change in *P* and *H* (d*P*/d*t* and d*H*/d*t*, respectively), as shown in figure 5*c*. We confirmed these findings by computing the cross-correlations *P*⊗*H* and d*P*/d*t*⊗d*H*/d*t* as a function of a lag between the two series (see §2). The graph in figure 5*d* clearly demonstrates the three principal findings: (*i*) there is a positive correlation between the level of serotype diversity and infection prevalence that is maximized at time-lag *Δ*_*P*_, (*ii*) the rate of change in diversity correlates inversely with the rate of change in disease prevalence, and (*iii*) the rate of change in diversity is positively correlated with prevalence at lag *Δ*_d*P*_. (This behaviour is also robust to changes in *ϕ* and *γ*, and can be found across a vast region of the enhancement spectrum; see the electronic supplementary material.) Taken together, these results strongly suggest an intricate interplay between the dynamics of individual serotypes and overall disease pattern of dengue where an increase in serotype diversity causes a subsequent increase in disease prevalence, which itself can cause a reduction in the number of circulating types.

## 4. Discussion

The limited amount of data available on the dynamics of co-circulating dengue serotypes suggest that they are complex, containing periods of synchronization between certain serotypes and desynchronization among others. Simple models of dengue transmission incorporating ADE have demonstrated that this behaviour is a natural feature of such a system (Ferguson *et al*. 1999; Cummings *et al*. 2005; Schwartz *et al*. 2005), provided that heterologous immunity is established upon secondary infection. However, it has recently been posited that ADE alone cannot account for the observed patterns, and that some form of cross-immunity after primary infection, in conjunction with other extrinsic factors, must be invoked to generate the appropriate periods and synchronization profiles (Adams *et al*. 2006). Here, we have shown that this limitation can be overcome by decomposing the effects of ADE on the population dynamics of dengue into two independent forms of enhancement: susceptibility and transmission. A wide range of dynamical regimes results from the disjunction between these two parameters, which easily accommodate the asynchrony of different serotypes and inter-epidemic periods of 3–5 years that the data evince. Furthermore, decomposing the effects of ADE significantly lowers the levels of enhancement necessary for obtaining realistic disease patterns. This has the added benefit of reducing the system's propensity for exhibiting the high amplitude oscillations, which in turn lowers the risk of stochastic extinction during the low-prevalence, inter-epidemic periods.

While our results suggest that ADE alone can give rise to observed patterns of serotype abundance through time, they do not rule out immunological cross-protection after primary infection as a contributing phenomenon. Indeed, immunological cross-protection through shared epitopes among four serotypes can give rise to periods of asynchrony (Recker *et al*. 2007), but not as reliably as through the action of ADE. Furthermore, the inclusion of a period of serotype-transcending immunity following primary infection can also cause desynchronized oscillations, even in the absence of ADE (Aguiar *et al*. 2008). However, in this case, the resulting periods in serotype prevalence and disease incidence are strongly coupled to the period of cross-immunity, a limitation that can easily be overcome by the inclusion of ADE. Another putative mechanism by which the dynamics of dengue can be explained has recently been suggested by Nagao & Koelle (2008); they conclude that a transient period of clinical cross-protection during which the hosts may gain immunity through heterologous infections without developing disease (together with seasonal variation in transmission) can account for observed epidemiological patterns, particularly the apparent shift towards longer periods in DHF incidence and in its age distribution. There is, however, no strong evidence as yet in favour of this mechanism.

There are a number of methods of discriminating between these various hypotheses. Certainly, further experimental studies on the effects of ADE are necessary to confirm its role of enhancing susceptibility and transmissibility within individuals suffering from severe disease during secondary infections. Its role in DHF has also yet to be clarified, although it is clear that excess mortality alone cannot account for observed dengue patterns (Wearing & Rohani 2006), but may play a role in the separation of serotypes (Kawaguchi *et al*. 2003). The extent of cross-protection conferred by primary infection can also be determined by experimental studies, and its role in the population dynamics of dengue may possibly be assessed by comparing patterns of dengue with other strain-structured pathogens where immunological cross-protection has been demonstrated. Similarly, the contribution of environmental factors will be better established as data emerge on patterns in areas of differing intensities and seasonalities of transmission. Spatial models and phylodynamic tools (as used in Adams *et al*. 2006) will play a critical role in connecting these processes to the patterns observed.

One avenue we have not yet explored is the possibility of asymmetries in the levels of enhancement (or cross-protection) between the four serotypes. For example, there is evidence that DENV-2 is more often associated with severe secondary infection, which suggests that it requires heterologous priming to cause severe disease (Sangkawibha *et al*. 1984; Anantapreecha *et al*. 2005). These asymmetries could also play an important role in terms of the sequence of infection, as shown by Endy *et al*. (2004). However, the inherent difficulty in determining infection histories from patient sera, due to high immunological cross-reactivity, prevents the construction of a coherent picture of serotype–serotype (immune) interaction. Future longitudinal cohort studies should help to illuminate the antigenic and genetic relationships between the circulating dengue serotypes. These will also help to elucidate the possible correlation between incidence of disease and serotype diversity identified by our model.

Finally, the enhancement of transmission as opposed to an increase in susceptibility may well be attributed to a different mechanism to ADE. A recent study shows DHF patients exhibit both a higher viral load and a slower rate of viral clearance in comparison with DF patients (Wang *et al*. 2006). For the latter, viral loads drop to undetectable levels by the first day of defervescence, but they remain at high levels in DHF patients. This difference in the rate of loss of viraemia is better explained by interference in the development of the appropriate immune response either by original antigenic sin or altered peptide ligand antagonism. If so, the two parameters in our model could represent two different positive interactions between serotypes, rather than two aspects of ADE alone. Whichever the biological basis, the decomposition into effects on susceptibility and transmission affords a parsimonious explanation for the observed variety in the epidemiological behaviour of dengue.

## Figures and Tables

**Figure 1:**
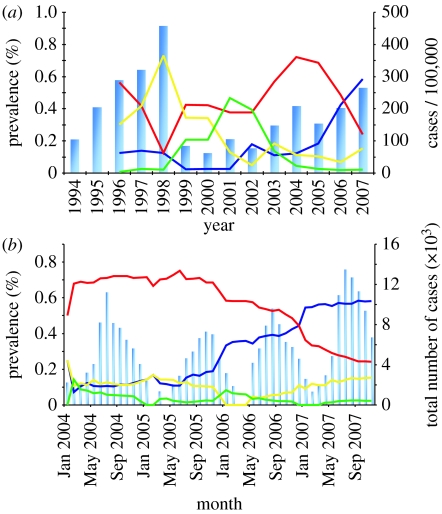
Dengue epidemiology in South Viet Nam. (*a*) The total annual number of dengue cases (blue bars) over the period 1994–2007 shows the characteristic fluctuation in disease incidence, whereas the relative serotype prevalence (1996–2007) clearly demonstrates the sequential replacements of dominant types (dark blue line, DEN-1; red line, DEN-2; yellow line, DEN-3; green line, DEN-4). (*b*) Monthly data of hospitalized dengue patients reveal a strong seasonal component in total disease burden (blue bars), which is not reflected in the relative frequency of the individual serotypes (dark blue line, DEN-1; red line, DEN-2; yellow line, DEN-3; green line, DEN-4).

**Figure 2:**
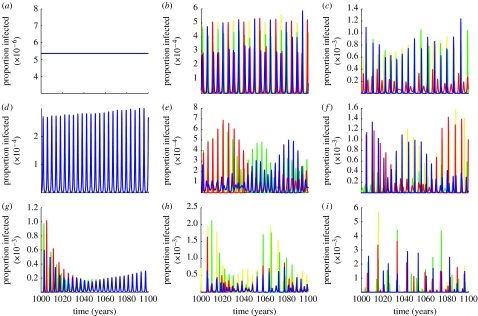
Model output showing the proportion of the population infected over time under changes of the degrees of enhancement. Each colour represents one of the four dengue serotypes. As the degree of enhancement increases (transmissibility enhancement from left to right and susceptibility enhancement from top to bottom), the system exhibits a rich catalogue of dynamical behaviours, from stable endemic equilibrium (*γ*=1, *φ*=1) to synchronized oscillations (e.g. *γ*=1, *φ*=1.9) and desynchronized and chaotic fluctuations with increased inter-epidemic periods (e.g. *γ*=1.8, *φ*=1.9). The inter-epidemic periods, *π*, also increase as the level of enhancement increases ((*a*) *π*=inf, (*b*) *π*=4*y*, (*c*) *π*=5*y*, (*d*) *π*=4*y*, (*e*) *π*=3*y*, (*f*) *π*=5*y*, (*g*) *π*=3*y*, (*h*) *π*=4*y* and (*i*) *π*=8*y*). Parameter values: (*a*) *φ*=1, *γ*=1; (*b*) *φ*=1.9, *γ*=1; (*c*) *φ*=2.4, *γ*=1; (*d*) *φ*=1, *γ*=2; (*e*) *φ*=1.9, γ=1.8; (*f*) *φ*=2.4, *γ*=1.7; (*g*) *φ*=1, *γ*=2.4; (*h*) *φ*=1.9, *γ*=2.4; and (*i*) *φ*=2.4, *γ*=2.4. Other parameter values: *β*=400, *σ*=100 and *μ*=1/70.

**Figure 3:**
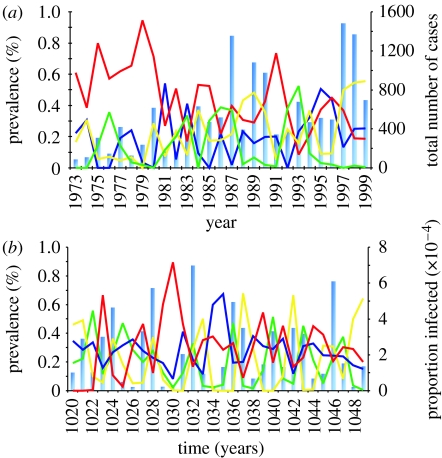
Qualitative comparison between model output and epidemiological data. (*a*) Data showing long-term record of total disease incidence (blue bars) and relative serotype prevalence in Thailand, taken from Nisalak *et al*. (2003) (dark blue line, DEN-1; red line, DEN-2; yellow line, DEN-3; green line, DEN-4). (*b*) Typical model outcome for a moderate degree of ADE (*γ*=2.1, *ϕ*=1.8) showing desynchronized oscillations in serotype prevalence and chaotic fluctuations in total disease prevalence (blue bars, arbitrary units; green line, serotype1; yellow line, serotype2; dark blue line, serotype3; red line, serotype4). Other parameter values: *β*=400, *σ*=100 and *μ*=1/70.

**Figure 4:**
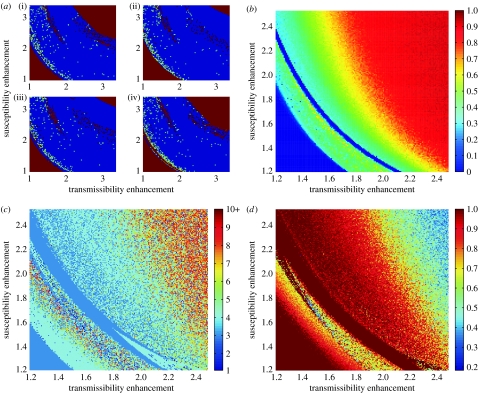
Analysis of the parameter space of enhancement. The (*ϕ*,*γ*)-parameter space can be qualified with respect to (*a*) synchronization patterns between two individual serotypes, (*b*) single-serotype prevalence, (*c*) major inter-epidemic period, and (*d*) serotype persistence. (*a*) The four synchronization patterns between serotypes (i) 1 and 2, (ii) 1 and 3, (iii) 2 and 3, and (iv) 3 and 4 reveal that the majority of the parameter space is occupied by desynchronized (blue) behaviour. Complete synchronization (red) indicates that after some initial transient, the time series of the two serotypes coincide and stay locked for the rest of the time interval; partial synchronization (green) is defined as a regime in which the time series are not fully synchronized, but throughout their 1000-year integration period have intervals of more than 100 years of synchronized behaviour. (*b*) Within the regime of desynchronized oscillation, we also find the system's behaviour being characterized by single-serotype replacement and dominance, where 0 indicates that at least two types are simultaneously dominant. (*c*) As the respective degrees of enhancement increase, we find the periods between epidemic outbreaks becoming longer, with a typical 3–5 year cycle occupying a large part of the parameter space and longer cycles (5–15 years) occurring towards the higher end of the enhancement spectrum. (*d*) The proportion of time a particular serotype persists above a persistence-threatening threshold level of 10^−8^ drops sharply as the level of enhancement increases, but remains high throughout a large region of the parameter space occupied by desynchronized, chaotic oscillations. Other parameter values: *β*=400, *σ*=100 and *μ*=1/70.

**Figure 5:**
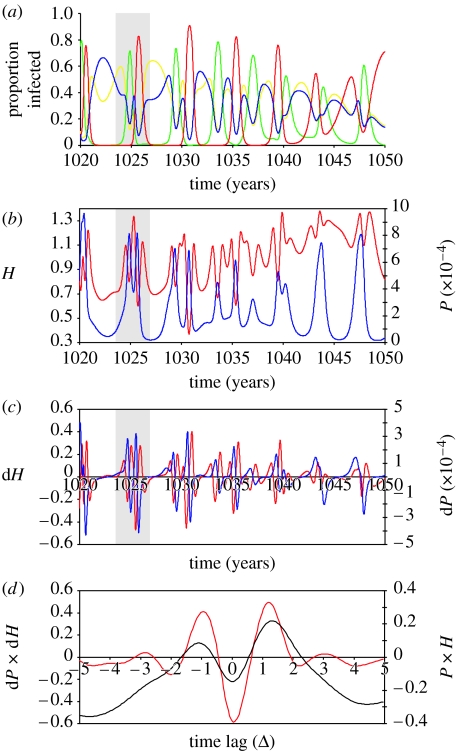
Cross-correlation analysis between serotype diversity and disease prevalence. (*a*) Time series of the relative frequency of the four dengue serotypes (red line, DEN-1; green line, DEN-2; yellow line, DEN-3; dark blue line, DEN-4) with (*b*) total disease prevalence (*P*, dark blue) and corresponding serotype diversity (*H*, red line); (*c*) the rates of change in prevalence and diversity, d*P*/d*t* and d*H*/d*t*, respectively. (*d*) The cross-correlation, *r*(*Δ*), between prevalence and diversity, *P*×*H* (black line), over a 100-year period reveals a bimodal distribution with two significant peaks at time lags *Δ*_*P*_ and *Δ*_−*P*_ and a ‘dip’ at lag *Δ*=0. This dip can be explained by the negative correlation at this point between the rates of change in prevalence and diversity, d*P*/d*t*×d*H*/d*t* (red line). The bimodal shape of the cross-correlation between the rates of change also reveals the negative feedback between prevalence and diversity. Parameter values: *γ*=2.1, *ϕ*=1.8, *β*=400, *σ*=100 and *μ*=1/70.

## Supplementary Material

supplementary figure 1 Figure showing the spectral analysis of the total proportion infected over time under changes of the degrees of enhancement

supplementary figure 2 Figure showing the cross-correlation between changes in diversity and prevalence across the parameter spectrum
